# Gender differences in comorbidities and risk factors in ischemic stroke patients with a history of atrial fibrillation

**DOI:** 10.1186/s12883-021-02214-8

**Published:** 2021-05-25

**Authors:** Chase Rathfoot, Camron Edrissi, Carolyn Breauna Sanders, Krista Knisely, Nicolas Poupore, Thomas Nathaniel

**Affiliations:** School of Medicine Greenville, University of South Carolina, Greenville, SC 29605 USA

**Keywords:** Acute ischemic stroke, Atrial fibrillation, Gender, Risk factors, Demographics

## Abstract

**Background:**

Atrial Fibrillation (AF) is a common cardiac arrhythmia and has been identified as a major risk factor for acute ischemic stroke (AIS). Gender differences in the disease process, causative mechanisms and outcomes of AF have been investigated. In the current study, we determined whether there is a gender-based disparity in AIS patients with baseline AF, and whether such a discrepancy is associated with specific risk factors and comorbidities.

**Methods:**

Baseline factors including comorbidities, risk and demographic factors associated with a gender difference were examined using retrospective data collected from a registry from January 2010 to June 2016 in a regional stroke center. Univariate analysis was used to differentiate between genders in terms of clinical risk factors and demographics. Variables in the univariate analysis were further analyzed using logistic regression. The adjusted odds ratios (ORs) and 95% confidence intervals (CIs) for each factor were used to predict the increasing odds of an association of a specific comorbidity and risk factor with the male or female AIS with AF.

**Results:**

In the population of AIS patients with AF, a history of drug and alcohol use (OR = 0.250, 95% CI, 0.497–1.006, *P* = 0.016), sleep apnea (OR = 0.321, 95% CI, 0.133–0.777, *P* = 0.012), and higher serum creatinine (OR = 0.693, 95% CI, 0.542–0.886 *P* = 0.003) levels were found to be significantly associated with the male gender. Higher levels of HDL-cholesterol (OR = 1.035, 95% CI, 1.020–1.050, *P* < 0.001), LDL-cholesterol (OR = 1.006, 95% CI, 1.001–1.011, *P* = 0.012), and the inability to ambulate on admission to hospital (OR = 2.258, 95% CI, 1.368–3.727, *P* = 0.001) were associated with females.

**Conclusion:**

Our findings reveal that in the AIS patients with atrial fibrillation, migraines, HDL, LDL and poor ambulation were associated with females, while drugs and alcohol, sleep apnea, and serum creatinine level were associated with male AIS patients with AF. Further studies are necessary to determine whether gender differences in risk factor profiles and commodities require consideration in clinical practice when it comes to AF as a risk factor management in AIS patients.

## Introduction

Atrial Fibrillation (AF) is a significant public health concern due to its growing prevalence and association with increased risk of cardiovascular events and death [1]. It is a common arrhythmia characterized by rapid, uncoordinated contraction of the atria, often resulting in thrombus formation and subsequent ischemia [2]. AF is a well-known independent risk factor in the development of acute ischemic stroke (AIS), as it is associated with a fivefold increase in the risk of stroke [3], and estimated to cause approximately one-fifth of all ischemic strokes, particularly of stronger severity and worse stroke-related outcomes [4]. Ischemic strokes in AF populations are associated with higher mortality, increased stroke recurrence, and greater functional deficits when compared to non-AF populations [5].

Although AF is less prevalent in females than in males, females with AF have an overall greater risk of stroke than males with AF [6, 7]. Studies also indicate that females with AF suffer from more severe strokes [8–10]. A recent study [8] found that within the AF population, male stroke patients presented with an average NIHSS score of 6 while female stroke patients had an average score of 9, indicating greater stroke severity and poorer functional outcomes among female stroke survivors. This finding is consistent with other studies that suggest that females with AF are more likely to suffer from debilitating or fatal strokes compared to males [3, 11, 12]. AF itself is a risk factor for stroke, we know that males have a higher incidence of AF at all age groups, while AF is less prevalent in females. However, females with AF have an overall greater risk of stroke than males with AF, suggesting that comorbidities and risk factors may be more pronounced in females. However, the risk factors and comorbidities associated with the observed gender difference in AIS with baseline AF are not clear. Since female AF patients are at higher risk of stroke severity and poor functional outcomes [13], it is possible that within the population of AIS patients with baseline AF, risk factors and comorbidities are not present in the same proportions. More risk factors and comorbidities maybe present among AF females presenting with stroke than among males. The first objective of this study is to identify the different risk factors and comorbidities in AIS population with AF and determine whether these risk factors and comorbidities are different between male and female AIS populations with a baseline AF. Since males and females do not present the same risk factors and comorbidities in the general AIS population, our second objective is to determine the association of males and females with the different comorbidities and risk factors using a retrospective data of AIS patients admitted between 2010 and 2016 to a primary stroke center. In this study, we analyzed risk factors, comorbidities and demographic variables to determine gender disparities in a stroke population with a baseline AF in a stroke center with an active patient protocol for the treatment of AIS population. Knowledge of these factors in males and females with AIS and baseline AF may necessitate different approaches to secondary prevention of stroke in patients with AF.

## Methods

### Study population

This is a population based cross sectional study for the retrospective analysis of patients’ data collected from PRISMA Health ischemic stroke population. This study was approved by the PRISMA Health Ethics Committee. PRISMA Health is a primary stroke center serving patients from an 8-county region located in upper South Carolina. Data for all patients treated between January 2010 and December 2016 in the Stroke Unit of PRISMA Health were used in this study. Stroke was defined according to the World Health Organization (WHO) criteria, as “a rapidly developing clinical symptoms of focal or global disturbance of cerebral function, lasting more than 24 h, with no apparent cause other than that of vascular origin” [14]. In this retrospective data analysis, data for ischemic stroke was based on neurologist assessment according to neuroimaging (including brain computed tomography scan and magnetic resonance imaging). Data for subtypes of stroke including subarachnoid and hemorrhagic stroke were excluded.

For each AIS patient, demographic information, risk factors, comorbidities or past medical history, and standard laboratory values were collected and analyzed. The registry has been described in previous retrospective studies [15–17]. For demographic data, information on age, race, gender, and ethnicity were retrieved in addition to their past medical and medication histories. This past medical history included data on atrial fibrillation, coronary artery disease (CAD), carotid stenosis, chronic renal disease (CRD), congestive heart failure (CHF), depression, diabetes mellitus, dyslipidemia, family history of stroke, hormone replacement therapy, hypertension, migraine, obesity, previous stroke or TIA, prosthetic heart valve, peripheral vascular disease (PVD), sleep apnea, substance use, and tobacco use. Additionally, data on site of admission (emergency department or direct admit) as well as ambulation were collected. Data on ambulation was recorded as either not documented (0), unable to ambulate (1), able to ambulate with assistance (2), and able to ambulate independently (3) at admission, during admission, and after discharge. Improvement was based on an increase in ambulation classification from admission to discharge.

### Data analysis

We conducted descriptive analyses to determine the distribution of the demographic data and risk factors in the AIS population using SPSS 24.0 (SPSS Inc. New York, New York, USA). All data have been previously normalized using the Shapiro–Wilk and Levene test to assess the data for homogeneity. Categorical variables were presented as proportions or expressed as frequencies (percentage) and quantitative variables as mean ± standard deviation. Variables were compared between male and female patients using Pearson x^2^ tests to identify demographic, comorbidities, risk factors, and laboratory values associated with males or females. For ordinal and dichotomous variables (such as ambulatory status or gender), a Pearson x^2^ test was used while a Student’s t-test was performed for all interval variables (such as age or BMI). A second univariate analysis was performed to differentiate between patients with and without a history of atrial fibrillation. Thereafter, three binary logistic multivariate analyses were performed; the first identified factors associated with gender in the whole AIS patients with and without AF. The dependent variable includes male or female patients. The second analysis focused on factors associated with males or females in the AIS population without AF, while the final analysis identified factors associated with males or females in the AIS population with baseline AF. These analyses were post-hoc adjusted logistic regression with a backward selection method. We used the backward approach because it has the advantage of considering the effects of all parameters simultaneously in fitting our regression models. This allows us to address the potential problem of multicollinearity by reducing the number of predictors and resolving the problem of overfitting. In the logistic regression, the association between the independent variables (the demographic information, comorbidities, risk factors, and laboratory values) and male or female (dependent variables) was determined for the atrial fibrillation and no atrial fibrillation group. The primary outcome is the adjusted odds ratios (ORs) and 95% confidence intervals (CIs) for each factor associated with each gender group, fully adjusting for all confounding factors identified in our univariate analyses. The resulting ORs were used to predict the increasing odds of an association of a specific factor with the male or female AIS with or without AF. A probability value of < 0.05 was considered statistically significant in the analyses. The Hosmer-Lemeshow test was used to validate our model, and the overall correct classification percentage including the area under the Receiver Operating Curve (ROC) for score prediction was determined to test the sensitivity, specificity and accuracy of our logistic model.

## Results

A total of 5469 AIS patients were identified. Of this, 2662 were males and 2807 were females. Table 1 presents the demographic and clinical characteristics of ischemic stroke patients stratified by male or female. As shown in Table 1, female patients were older (69.26 ± 15.657 vs 65.13 ± 13.372) and presented with a significantly higher BMI (28.61 ± 7.795 vs 28.04 ± 5.983) than the male patients. For past medical history, female patients presented with significantly higher rates of atrial fibrillation (19.3% vs 14.3%), depression (16.0% vs 10.2), heart failure (12.3% vs 9.2%), hypertension (80.2% vs 77.2%), and migraines (3.7% vs 1.1%). On the other hand, male patients were more likely to present with a history of coronary artery disease (35.1% vs 25.9%), carotid artery stenosis (7.2 to 5.1%), sleep apnea (3.8% vs 2.5%), substance use (9.6% vs 2.9%), and tobacco use (33.2% vs 21.5%). Regarding medication history, females were more likely to be on antihypertensives and antidepressants (72.4% vs 66.2%; 16.4% vs 9.4%) while males were more likely to be on cholesterol reducers (46.1% vs 42.8%). Initial laboratory values also differed between male and female patients. Male patients presented with significantly higher levels of serum creatinine (1.40 ± 1.22 vs 1.18 ± 1.09) whereas female patients had higher total cholesterol (177.54 ± 50.89 mg/dL vs. 165.96 ± 52.10 mg/dL), HDL-cholesterol (45.03 ± 14.23 mg/dL vs. 38.43 ± 12.57 mg/dL), and LDL-cholesterol (107.31 ± 42.58 mg/dL vs. 101.83 ± 39.72 mg/dL). Female patients also presented with a higher heart rate (83.50 ± 18.57 vs 80.43 ± 15.35) and a lower diastolic blood pressure (80.39 ± 19.59 vs 84.60 ± 18.37). Finally, a significant gender difference was observed in ambulation and stroke severity with female patients presenting with a less improved ambulation (34.3 to 37.5%) and higher NIHSS scores (42.1% vs 35.0%) when compared to males.

**Table 1:** Demographic and clinical characteristics of acute ischemic stroke patients compared by gender. Results for continuous variables are presented as Mean ± SD, while discrete data are presented as percentage and frequency. Student T-test and Pearson’s Chi-Square were used

Characteristic	Male	Female	
Number of patients	2662	2807	***P-*value**
Age Group: No. (%)			
< 50	305 (11.5)	353 (12.6)	< 0.001*^a^
50–59	603 (22.7)	393 (14.0)	
60–69	740 (27.8)	559 (19.9)	
70–79	592 (22.2)	639 (22.8)	
> =80	422 (15.9)	30.7)	
Mean ± SD	65.13 ± 13.372	69.26 ± 15.657	< 0.001*^b^
Race: No (%)			
White	2090 (78.5)	2198 (78.3)	0.544
Black	492 (18.5)	510 (18.2)	
Other	80 (3.0)	99 (3.5)	
Hispanic Ethnicity: No. (%)	38 (1.4)	47 (1.7)	0.461
BMI: Mean ± SD	28.04 ± 5.983	28.61 ± 7.795	0.002*^b^
Medical History: No. (%)			
Atrial Fib	381 (14.3)	543 (19.3)	< 0.001*^a^
Coronary Artery Disease	935 (35.1)	726 (25.9)	< 0.001*^a^
Carotid Artery Stenosis	191 (7.2)	143 (5.1)	0.001*^a^
Depression	271 (10.2)	450 (16.0)	< 0.001*^a^
Diabetes	951 (35.7)	984 (35.1)	0.605
Drugs or Alcohol	256 (9.6)	81 (2.9)	< 0.001*^a^
Dyslipidemia	1364 (51.2)	1391 (49.6)	0.213
Stroke Family History	224 (8.4)	270 (9.6)	0.121
Heart Failure	245 (9.2)	345 (12.3)	< 0.001*^a^
Hypertension	2056 (77.2)	2250 (80.2)	0.008*^a^
Migraine	29 (1.1)	105 (3.7)	< 0.001*^a^
Obesity	1148 (43.1)	1163 (41.4)	0.205
Previous Stroke	671 (25.2)	753 (26.8)	0.173
Previous TIA (> 24 h)	213 (8.0)	264 (9.4)	0.066
Prosthetic Heart Valve	35 (1.3)	27 (1.0)	0.218
Peripheral Vascular Disease	192 (7.2)	208 (7.4)	0.779
Chronic Renal Disease	232 (8.7)	215 (7.7)	0.154
Sleep Apnea	101 (3.8)	69 (2.5)	0.004*^a^
Smoker	883 (33.2)	603 (21.5)	< 0.001*^a^
Medication History: No (%)			
HTN medication	1763 (66.2)	2031 (72.4)	< 0.001*^a^
Cholesterol Reducer	1227 (46.1)	1201 (42.8)	0.014*^a^
Diabetic Medication	735 (27.6)	760 (27.1)	0.657
Antidepressant	251 (9.4)	460 (16.4)	< 0.001*^a^
Initial NIHSS Score: No (%)			
0–9	1677 (74.0)	1612 (69.5)	0.005*^a^
10–14	235 (10.4)	272 (11.7)	
15–20	218 (9.6)	283 (12.2)	
21–25	137 (6.0)	153 (6.6)	
Mean ± SD	7.63 ± 7.84	8.90 ± 8.56	< 0.001*^b^
Lab values: Mean ± SD			
Total cholesterol	165.96 ± 52.10	177.54 ± 50.89	< 0.001*^b^
Triglycerides	142.60 ± 110.95	136.78 ± 99.16	0.057
HDL	38.43 ± 12.57	45.03 ± 14.23	< 0.001*^b^
LDL	101.83 ± 39.72	107.31 ± 42.58	< 0.001*^b^
Lipids	6.51 ± 1.82	6.54 ± 3.11	0.709
Blood Glucose	146.75 ± 78.26	147.82 ± 83.62	0.626
Serum Creatinine	1.40 ± 1.22	1.18 ± 1.09	< 0.001*^b^
INR	1.15 ± 0.51	1.18 ± 1.09	0.069
Vital Signs: Mean ± SD			
Heart Rate	80.43 ± 15.35	83.50 ± 18.57	< 0.001*^b^
Blood Pressure Systolic	152.09 ± 28.86	151.57 ± 29.74	0.508
Blood Pressure Diastolic	84.60 ± 18.37	80.39 ± 19.59	< 0.001*^b^
Ambulation Status Prior to Event: No. (%)			
Ambulate Independently	2453 (92.1)	2434 (86.7)	< 0.001*^a^
Ambulate with Assistance	74 (2.8)	129 (4.6)	
Unable to Ambulate	79 (3.0)	134 (4.8)	
Not Documented	56 (2.1)	109 (3.9)	
Ambulation Status on Admission: No. (%)			
Ambulate Independently	727 (27.3)	604 (21.5)	< 0.001*^a^
Ambulate with Assistance	790 (29.7)	836 (29.8)	
Unable to Ambulate	744 (27.9)	984 (35.1)	
Not Documented	401 (15.1)	383 (13.6)	
Ambulation Status on Discharge: No. (%)			
Ambulate Independently	1184 (44.5)	990 (35.3)	< 0.001*^a^
Ambulate with Assistance	846 (31.8)	974 (34.7)	
Unable to Ambulate	429 (16.1)	640 (22.8)	
Not Documented	203 (7.6)	203 (7.2)	
rtPA received: No. (%)	668 (25.1)	659 (23.5)	0.163
First Care Received: No. (%)			
Emergency Department	2085 (79.1)	2212 (79.4)	0.806
Direct Admission	550 (20.9)	574 (20.6)	
Improved Ambulation: No. (%)	927 (37.5)	898 (34.4)	0.019*^a^
NIHSS > 7: No. (%)	824 (35.0)	1043 (42.1)	< 0.001*^a^
Diastolic Blood Pressure ≥ 80 mmHg	1576 (59.3)	1312 (46.8)	< 0.001*^a^

Notes:

^a^Pearson’s Chi-Squared test

^b^Student’s T test

* *P*-value < 0.05

Table 2 presents demographic and clinical characteristics of male and AIS patients stratified by the presence or absence of AF. In the AIS with no AF group, females presented with a higher age and BMI (66.66 ± 15.581 vs 63.65 ± 13.156; 29.084 ± 28.1229 ± 6.041); higher rates of depression (16.6% vs 9.9%), hormone replacement therapy (2.8% vs 0.0%), migraines (4.2% vs 1.2%), previous TIAs (9.5% vs 7.7%); and higher laboratory values such as total cholesterol (181.17 ± 51.94 vs 168.81 ± 46.97), HDL (44.94 ± 14.44 vs 38.45 ± 12.60), and LDL (110.03 ± 43.69 vs 104.33 ± 40.11).In addition, females presented with a higher rate for the use of antihypertensive and antidepressants (68.3% vs 62.6%; 16.6% vs 9.2%), and with a higher mean heart rate (83.21 ± 18.03 vs 80.20 ± 18.10). Male patients without atrial fibrillation presented with a higher rate of coronary artery disease (32.1% vs 23.0%), substance (10.4% vs 3.3%), and tobacco use (36.6% vs 25%), higher serum creatine level (1.39 ± 1.21 vs 1.18 ± 1.17), INR (1.09 ± 0.29 vs 1.07 ± 0.28), diastolic blood pressure (85.23 ± 18.26 vs 80.26 ± 19.36) and ambulation improvement.

**Table 2:** Demographic and clinical characteristics of compared between atrial fibrillation status in ischemic stroke patients stratified by gender. Results for continuous variables are presented as Mean ± SD, while discrete data are presented as percentage frequency. Student T-test and Pearson’s Chi-Square were used

	No Atrial Fibrillation			With Atrial Fibrillation		
Characteristic	Male	Female		Male	Female	
Number of patients	2281	2264	*P*-value	381	543	*P*-Value
Age Group: No. (%)						
< 50 years	295 (12.9)	348 (15.4)	< 0.001* ^a^	10 (2.6)	5 (0.9)	< 0.001* ^a^
50–59	568 (24.9)	374 (16.5)		35 (9.2)	19 (3.5)	
60–69	666 (29.2)	499 (22.0)		74 (19.4)	60 (11.0)	
70–79	468 (20.5)	510 (22.5)		124 (32.5)	129 (23.8)	
> =80	284 (12.5)	533 (23.5)		138 (36.2)	330 (60.8)	
Age Mean ± SD	63.65 ± 13.156	66.66 ± 15.581	< 0.001* ^b^	74.01 ± 11.013	80.09 ± 10.475	< 0.001* ^b^
Race: No (%)						
White	1753 (76.9)	1728 (76.3)	0.425	337 (88.5)	470 (86.6)	0.692
Black	452 (19.8)	444 (19.6)		40 (10.5)	66 (12.2)	
Other	76 (3.3)	92 (4.1)		4 (1.0)	7 (1.3)	
Hispanic Ethnicity: No. (%)	37 (1.6)	41 (1.8)	0.624	1 (0.3)	6 (1.1)	0.146
BMI: Mean ± SD	28.1229 ± 6.041	29.084 ± 7.847	< 0.001* ^b^	27.578 ± 5.619	26.669 ± 7.263	0.034* ^b^
Medical History: No. (%)						
Coronary Artery Disease	732 (32.1)	521 (23.0)	< 0.001* ^a^	203 (53.3)	205 (37.8)	< 0.001* ^a^
Carotid Artery Stenosis	152 (6.7)	120 (5.3)	0.053	39 (10.2)	23 (4.2)	< 0.001* ^a^
Depression	226 (9.9)	375 (16.6)	< 0.001* ^a^	45 (11.8)	75 (13.8)	0.373
Diabetes	798 (35.0)	817 (36.1)	0.438	153 (40.2)	167 (30.8)	0.003* ^a^
Drugs or Alcohol	238 (10.4)	75 (3.3)	< 0.001* ^a^	18 (4.7)	6 (1.1)	0.001* ^a^
Dyslipidemia	1112 (48.8)	1104 (48.8)	0.993	252 (66.1)	287 (52.9)	< 0.001
Stroke Family History	190 (8.3)	216 (9.5)	0.152	34 (8.9)	54 (9.9)	0.603
Heart Failure	156 (6.8)	187 (8.3)	0.070	89 (23.4)	158 (29.1)	0.052
HRT	1 (0.0)	64 (2.8)	< 0.001* ^a^	0 (0.0)	14 (2.6)	0.002* ^a^
Hypertension	1727 (75.7)	1763 (77.9)	0.085	329 (86.4)	487 (89.7)	0.120
Migraine	28 (1.2)	95 (4.2)	< 0.001* ^a^	1 (0.3)	10 (1.8)	0.029* ^a^
Obesity	982 (43.1)	992 (43.8)	0.603	166 (43.6)	171 (31.5)	< 0.001* ^a^
Previous Stroke	559 (24.5)	590 (26.1)	0.228	112 (29.4)	163 (30.0)	0.839
Previous TIA (> 24 h)	175 (7.7)	214 (9.5)	0.032* ^a^	38 (10.0)	50 (9.2)	0.696
Prosthetic Heart Valve	22 (1.0)	16 (0.7)	0.340	13 (3.4)	11 (2.0)	0.192
Peripheral Vascular Disease	153 (6.7)	154 (6.8)	0.899	39 (10.2)	54 (9.9)	0.885
Chronic Renal Disease	184 (8.1)	160 (7.1)	0.203	48 (12.6)	55 (10.1)	0.240
Sleep Apnea	77 (3.4)	55 (2.4)	0.57	24 (6.3)	14 (2.6)	0.005* ^a^
Smoker	834 (36.6)	567 (25.0)	< 0.001* ^a^	49 (12.9)	36 (6.6)	0.001* ^a^
HTN medication	1428 (62.6)	1546 (68.3)	< 0.001* ^a^	335 (87.9)	485 (89.3)	0.510
Cholesterol Reducer	989 (43.4)	951 (42.0)	0.357	238 (62.5)	250 (46.0)	< 0.001* ^a^
Diabetic Medication	615 (27.0)	644 (28.4)	0.264	120 (31.5)	116 (21.4)	0.001* ^a^
Antidepressant	209 (9.2)	375 (16.6)	< 0.001* ^a^	42 (11.0)	85 (15.7)	0.044* ^a^
Lab values: Mean ± SD						
Total cholesterol	168.81 ± 46.974	181.17 ± 51.94	< 0.001* ^b^	148.72 ± 73.92	161.76 ± 42.702	0.002
Triglycerides	146.89 ± 114.017	142.5 ± 103.031	0.205	116.65 ± 85.91	111.91 ± 75.410	0.414
HDL	38.45 ± 12.601	44.94 ± 14.437	< 0.001* ^b^	38.33 ± 12.396	45.39 ± 13.337	< 0.001* ^b^
LDL	104.33 ± 40.106	110.03 ± 43.688	< 0.001* ^b^	86.78 ± 33.636	95.52 ± 35.111	0.001* ^b^
Lipids	6.5401 ± 1.86633	6.6506 ± 3.3761	0.209	6.3937 ± 1.518	6.0918 ± 1.3526	0.005* ^b^
Blood Glucose	147.41 ± 80.058	150.25 ± 87.482	0.254	142.83 ± 66.47	137.65 ± 64.136	0.239
Serum Creatinine	1.3927 ± 1.21170	1.1840 ± 1.1746	< 0.001* ^b^	1.501 ± 1.3264	1.1714 ± 0.70412	< 0.001* ^b^
INR	1.0963 ± 0.29154	1.0714 ± 0.2832	0.010* ^b^	1.4824 ± 1.049	1.3406 ± 0.8496	0.042* ^b^
Vital Signs: Mean ± SD						
Heart Rate	80.20 ± 18.099	83.21 ± 18.034	< 0.001* ^b^	81.79 ± 19.790	84.72 ± 20.642	0.031* ^b^
Blood Pressure Systolic	152.82 ± 29.094	151.69 ± 30.417	0.204	147.77 ± 27.06	151.04 ± 26.749	0.069
Blood Pressure Diastolic	85.23 ± 18.257	80.26 ± 19.358	< 0.001* ^b^	80.84 ± 18.633	80.94 ± 20.540	0.939
Ambulation Status Prior to Event: No. (%)						
Ambulate Independently	2119 (92.9)	2033 (89.8)	0.003* ^a^	334 (87.7)	401 (73.8)	< 0.001* ^a^
Ambulate with Assistance	58 (2.5)	79 (3.5)		16 (4.2)	50 (9.2)	
Unable to Ambulate	65 (2.8)	95 (4.2)		14 (3.7)	39 (7.2)	
Not Documented	39 (1.7)	57 (2.5)		17 (4.5)	52 (9.6)	
Ambulation Status on Admission: No. (%)						
Ambulate Independently	660 (28.9)	542 (23.9)	< 0.001* ^a^	67 (17.6)	62 (11.4)	< 0.001* ^a^
Ambulate with Assistance	671 (29.4)	722 (31.9)		119 (31.2)	114 (21.0)	
Unable to Ambulate	611 (26.8)	686 (30.3)		133 (34.9)	298 (54.9)	
Not Documented	339 (14.9)	314 (13.9)		62 (16.3)	69 (12.7)	
Ambulation Status on Discharge: No. (%)						
Ambulate Independently	1061 (46.5)	887 (39.2)	< 0.001* ^a^	123 (32.3)	103 (19.0)	< 0.001* ^a^
Ambulate with Assistance	718 (31.5)	796 (35.2)		128 (33.6)	178 (32.8)	
Unable to Ambulate	338 (14.8)	449 (19.8)		91 (23.9)	191 (35.2)	
Not Documented	164 (7.2)	132 (5.8)		39 (10.2)	71 (13.1)	
rtPA Administration	584 (25.6)	532 (23.5)				
First Care Received No. (%)						
Emergency Department	1772 (78.5)	1767 (78.7)	0.895	313 (82.8)	445 (82.4)	0.876
Direct Admission	485 (21.5)	479 (21.3)		65 (17.2)	95 (17.6)	

Notes:

^a^Pearson’s Chi-Squared test

^b^Student’s T test

* *P*-value < 0.05

For AIS patients with a past medical history of AF, females were older (80.09 ± 10.48 vs 74.01 ± 11.01), use antidepressants (15.7% vs 11.0%), HDL-cholesterol (45.39 ± 13.34 vs 38.33 ± 12.40), and presents with higher HRT (2.6% vs 0.0%), migraines (1.8% vs 0.3%), LDL-cholesterol (95.52 ± 35.11 vs 86.78 ± 33.64), and heart rate (84.72 ± 20.64 vs 81.79 ± 19.79). Male AIS patients with AF were more likely to present with higher BMI (27.58 ± 5.62 vs 26.67 ± 7.26), a past medical history of coronary artery disease (53.3% vs 37.8%), carotid artery stenosis (10.2% vs 4.2%), diabetes (40.2% vs 30.8%), substance use (4.7% vs 1.1%), obesity (43.6% vs 31.5%), sleep apnea (6.3% vs 2.6%), tobacco use (12.9% vs 6.6%), cholesterol reducers (62.5% vs 46.0%), and diabetic medication (31.5% vs 21.4%); and higher laboratory values including serum creatinine (1.50 ± 1.33 vs 1.17 ± 0.70) and INR (1.48 ± 1.05 vs 1.34 ± 0.85).

**Fig. 1:**
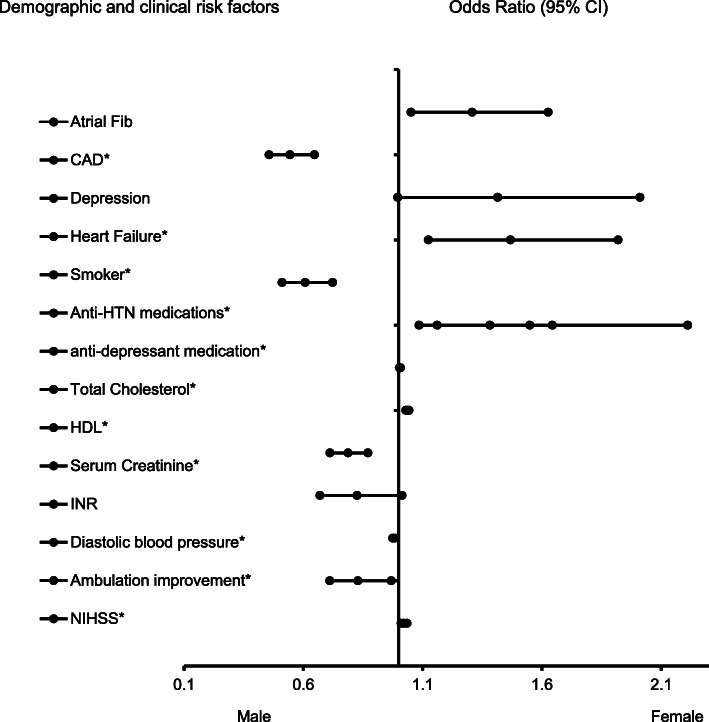
Forest Plot representation for clinical and demographic factors associated with ischemic stroke patients with and without atrial fibrillation. Adjusted OR < 1 denotes factors that are associated with male while OR > 1 denote factors that are associated with females. Hosmer-Lemeshow test (*P* = 0.546), Cox & Snell *(R*^*2*^ = 0.149) were analyzed. The overall classified percentage of 67.1% was applied to check for fitness of the logistic regression model. *Indicates statistical significance (*P* < 0.05) with a 95% confidence interval. ^Indicates that data were modified by taking the 5th square root for graphing purposes

**Fig. 2:**
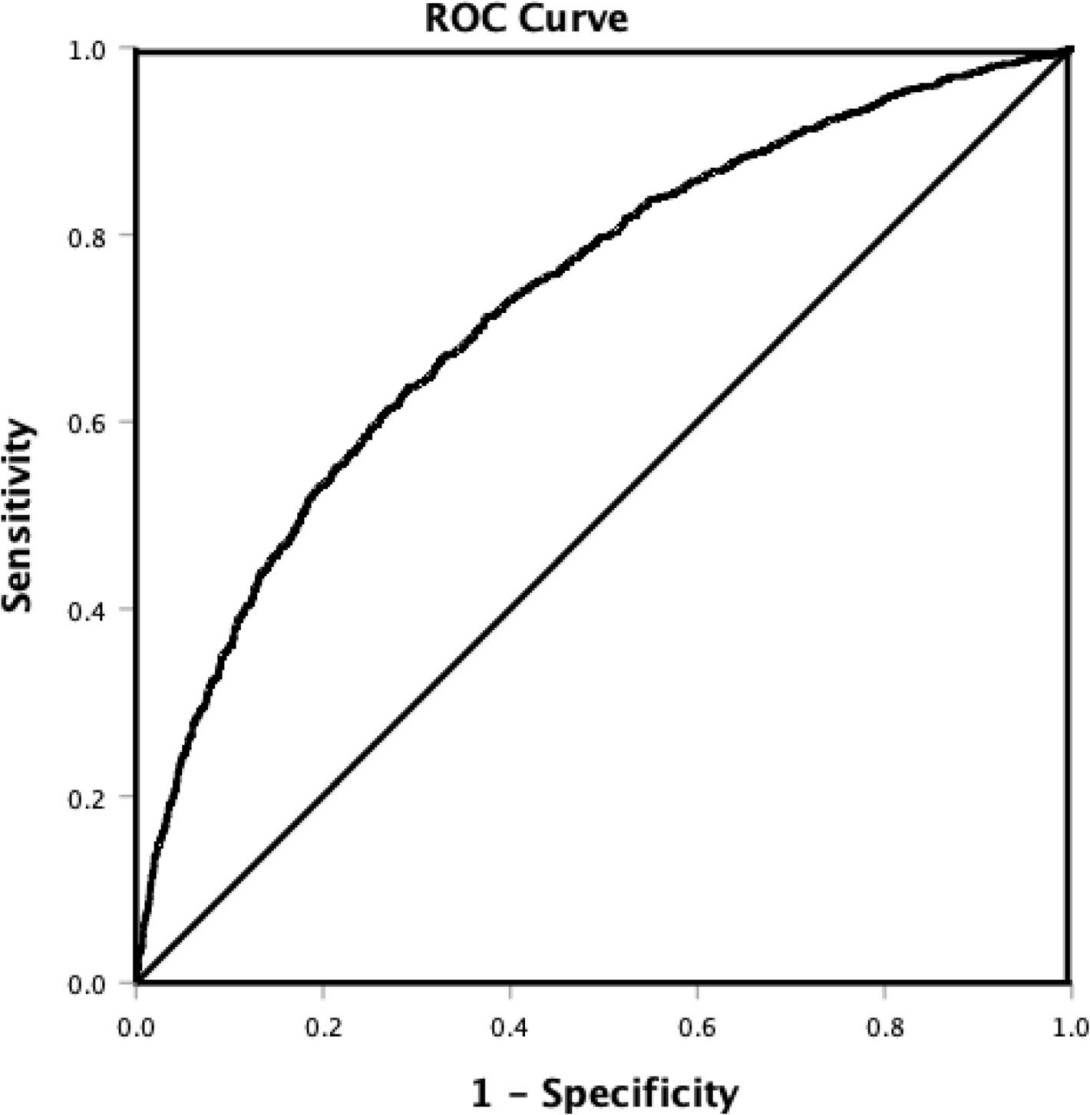
ROC curve associated with acute ischemic stroke patients with and without atrial fibrillation. Elevated area under the curve (AUC) values in ROC analysis indicate stronger discrimination of the score for being female. ROC curve (AUC = 0.729, 0.712–0.746) was used to analyze sensitivity and specificity of the model

In the adjusted analysis for the whole AIS population with and without AF (Fig. 1), coronary artery disease (OR = 0.544, 95% CI, 0.457–0.65, *P* < 0.001), history of smoking (OR = 0.608, 95% CI, 0.511–0.72, P < 0.001), increasing serum creatinine (OR = 0.788, 95% CI, 0.712–0.87, *P* < 0.001), increasing diastolic blood pressure (OR = 0.979, 95% CI, 0.975–0.98, *P* = 0.001), changes or improvement in ambulation (OR = 0.829, 95% CI, 0.711–0.97, *P* = 0.017) were associated with males, while atrial fibrillation (OR = 1.308, 95% CI, 1.051–1.63, *P* = 0.016), heart failure (OR = 1.468, 95% CI, 1.124–1.92, *P* = 0.005), anti-HTN medication (OR = 1.549, 95% CI, 1.085–2.21, P = 0.016), increasing total cholesterol l(OR = 1.005, 95% CI, 1.003–1.01, *P* < 0.001), increasing HDL-cholesterol (OR = 1.037, 95% CI, 1.03–1.04, P < 0.001), and increasing NIHSS (OR = 1.022, 95% CI, 1.01–1.04, *P* < 0.001) were associated with females. As shown in Fig. 2, the ROC Curve (AUC = 0.729, 95% CI, 0.7112–0.746, *P* < 0.001) demonstrates a strong sensitivity and specificity of our regression model.

**Fig. 3:**
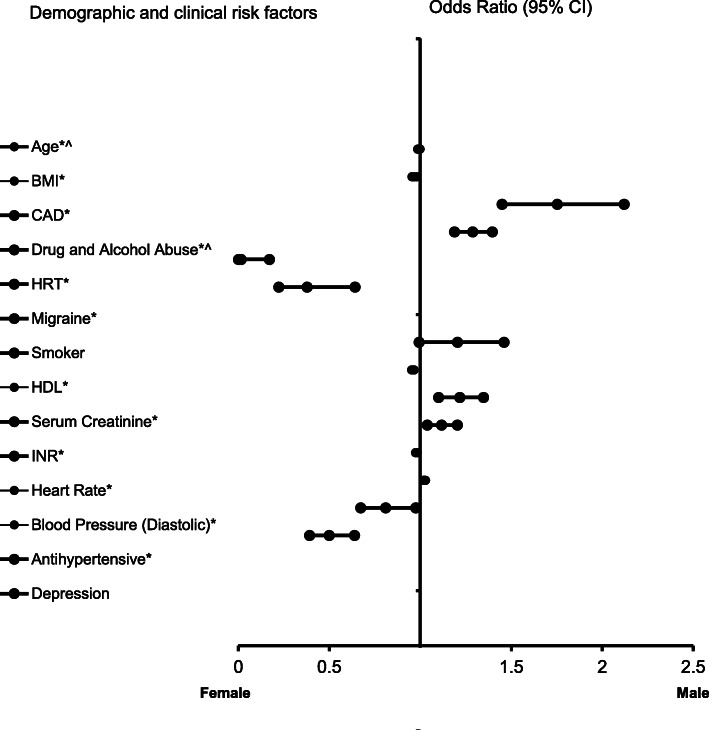
Forest Plot representation for clinical factors associated with ischemic stroke patients without atrial fibrillation. Adjusted OR < 1 denote factors that are associated with females while OR > 1 denote factors that are associated with males. Hosmer-Lemeshow test (*P* = 0.866), Cox & Snell *(R*^*2*^ *= 0.189*) were analyzed. The overall classified percentage of 70.0% was applied to check for fitness of the logistic regression model. *Indicates statistical significance (*P* < 0.05) with a 95% confidence interval. ^Indicates that data were modified by taking the 5th square root for graphing purposes

**Fig. 4:**
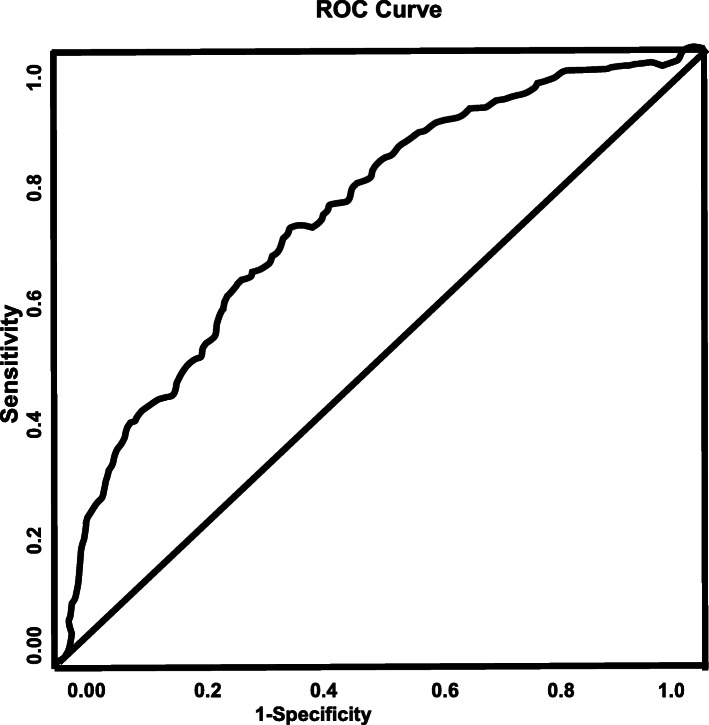
ROC curve associated with acute ischemic stroke patients without atrial fibrillation. Area under the curve (AUC) values in ROC analysis indicate better discrimination of the score for being male. ROC curve (AUC = 0.757, 0.740–0.774) was used to analyze sensitivity and specificity of the model

In the adjusted analysis for the AIS patients without AF, increasing age (OR = 0.992, 95% CI, 0.986–0.999, *P* = 0.032), BMI (OR = 0.968, 95% CI, 0.955–0.981, P < 0.001), depression (OR = 0.500, 95% CI, 0.392–0.639), HRT (OR = 0.016, 95% CI, 0.001–0.171, *P* = 0.001), migraine (OR = 0.379, 95% CI, 0.223–0.642, P < 0.001), increasing HDL-cholesterol (OR = 0.959, 95% CI, 0.953–0.966, P < 0.001), increasing heart rate (OR = 0.980, 95% CI, 0.975–0.985, *P* < 0.001), and antihypertensive (OR = 0.810, 95% CI, 0.673–0.976, *P* = 0.026) were associated with females without AF, while CAD (OR = 1.754, 95% CI, 1.450–2.121, *P* < 0.0001), drugs and alcohol (OR = 3.560, 95% CI, 2.379–5.327, P < 0.0001), higher serum creatinine level (OR = 1.218, 95% CI, 1.101–1.348, P < 0.0001), INR(OR = 1.749, 95% CI, 1.208–2.532, *P* = 0.003), and increasing diastolic blood pressure (OR = 1.024, 95% CI, 1.019–1.029, P < 0.0001), were associated with males (Fig. 3). The ROC Curve (AUC = 0.757, 95% CI, 0.740–0.774, *P* < 0.001) demonstrates a strong sensitivity and specificity of the model (Fig. 4).

**Fig. 5:**
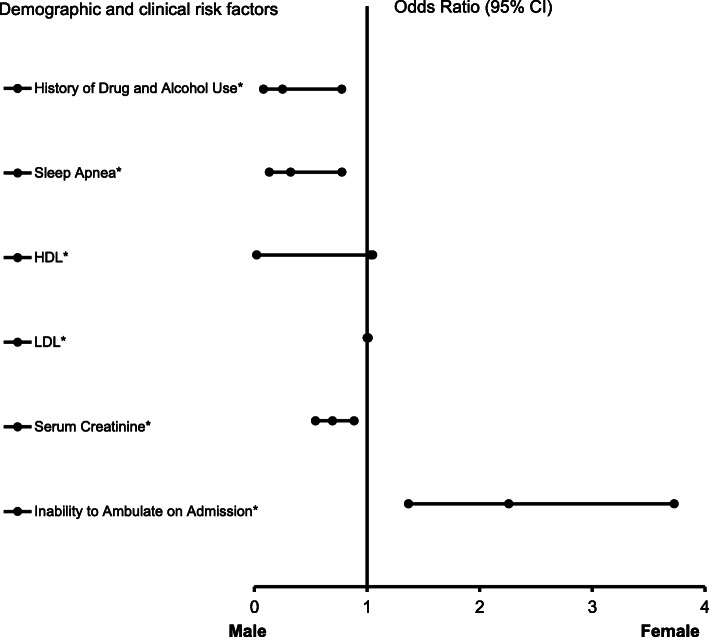
Forest Plot representation for clinical factors associated with ischemic stroke patients with atrial fibrillation. Adjusted OR < 1 denote factors that are associated with males while OR > 1 denote factors that are associated with females. Hosmer-Lemeshow test (*P* = 0.866), Cox & Snell *(R*^*2*^ *= 0.189*) were analyzed. The overall classified percentage of 70.0% was applied to check for fitness of the logistic regression model. *Indicates statistical significance (*P* < 0.05) with a 95% confidence interval. ^Indicates that data were modified by taking the 5th square root for graphing purposes

**Fig. 6:**
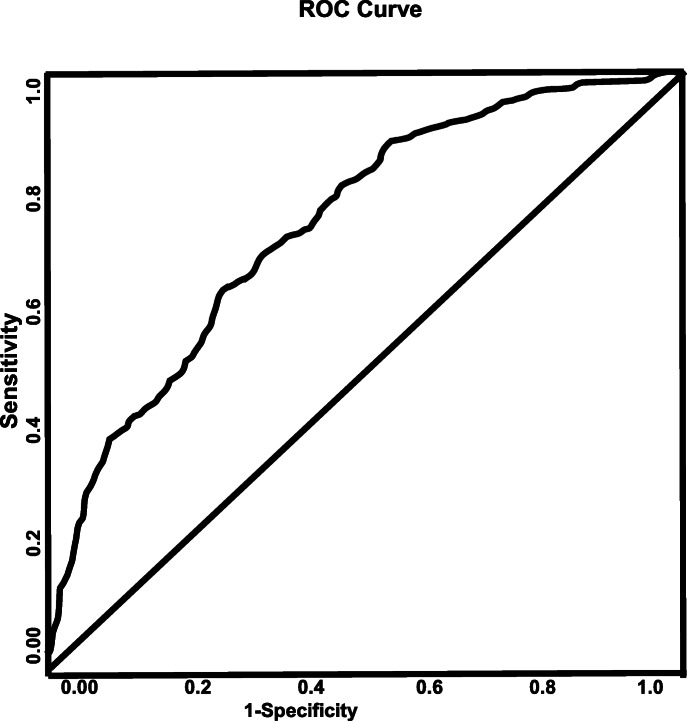
ROC curve associated with acute ischemic stroke patients with atrial fibrillation. Area under the curve (AUC) values in ROC analysis indicate better discrimination of the score for being female. ROC curve (AUC = 0.757, 0.721–0.793) was used to analyze sensitivity and specificity of the model

In the AIS patients with atrial fibrillation (Fig. 5), migraines (OR = 10.748, 95% CI, 0.954–121.135, *P* = 0.016), increasing HDL (OR = 1.035, 95% CI, 1.020–1.050, *P* < 0.001), increasing LDL-cholesterol (1.006, 95% CI, 1.001–1.011, *P* = 0.003), and the inability to ambulate on admission (OR = 2.258, 95% CI, 1.368–3.727, *P* = 0.001) were associated with females, while history of drug and alcohol abuse (OR = 0.250, 95% CI, 0.081–0.776, P = 0.016), sleep apnea (OR = 0.321, 95% CI, 0.133–0.777, *P* = 0.012), and increasing serum creatinine level (OR = 0.693, 95% CI, 0.542–0.886, P = 0.003) were associated with males. The ROC curve (Fig. 6) demonstrates the sensitivity and specificity of the model to be strong with an AUC of 0.757, 95% CI, 0.721–0.793, and *P* < 0.001).

## Discussion

Three major findings arise from this study. First, we found that in the whole AIS population with and without AF, coronary artery disease, history of smoking, increasing serum creatinine, increasing diastolic blood pressure, changes or improvement in ambulation, were associated with males, while atrial fibrillation, heart failure, anti-HTN medication, increasing total cholesterol, increasing HDL-cholesterol and increasing NIHSS, were associated with females. Second, in AIS patients without AF, increasing age, BMI, depression, HRT, migraine, increasing HDL-cholesterol, increasing heart rate, and antihypertensive were associated with females without AF, while CAD, drugs and alcohol, higher serum creatinine level, INR, and increasing diastolic blood pressure were associated with males. Finally, in the AIS patients with AF, migraines, increasing HDL, LDL-cholesterol, and the inability to ambulate on admission were associated with females, while history of drug and alcohol abuse, sleep apnea, and increasing serum creatinine level were associated with males.

The effect of HDL-cholesterol which was significant in the whole AIS with and without AF was sustained in the adjusted analysis for the female AIS patients without AF. Although age, BMI, depression, HRT, migraine, heart rate, and antihypertensive medications were not associated with female patients in the whole AIS population, these factors were significant and associated with females in the adjusted analysis for the AIS patients without AF. The effect of coronary artery disease, smoking and higher rates of improved ambulation which were more likely to be associated with male patients in the whole AIS with and without AF were attenuated in male patients for the adjusted analysis of AIS patients without AF. In addition, higher serum creatinine levels and diastolic blood pressure were significant among males in the whole AIS with and without AF, and such an effect was sustained and associated with male AIS patients without AF in the adjusted analysis. While CAD, drugs and alcohol were not significant in the whole AIS population, they were significantly associated with males in the adjusted analysis for the AIS without AF.

Our finding that in the AIS population without AF, older AIS patients that present with depression, HRT, migraine, elevated HDL-cholesterol, elevated heart rate, and take antihypertensive medications were associated with females, while CAD, history of drugs and alcohol abuse, high serum creatinine level, elevated INR and increased diastolic blood pressure were associated with males are consistent with other studies for both females [18–21] and for males [22–26]. Findings indicate that female AIS patients present a worse prognosis than men overall [27]. Precisely, hypertension and use of antihypertensive medications were more frequent in females than in males [21, 28]. The effect of depression [29], HRT [30], migraine [31], HDL [32] and heart rate [33] were more severe in females, while CAD [34], drugs and alcohol use [35], high serum creatinine level [36], INR [25] and diastolic blood pressure [26] were more prevalent in male AIS patients.

We observed that in AIS patients with atrial fibrillation, migraines, elevated HDL-cholesterol and LDL-cholesterol with the inability to ambulate on admission were associated with females, while history of drug and alcohol abuse, sleep apnea, and higher serum creatinine level were associated with male AIS patients with AF. Our finding of the association of poor ambulatory outcome with female AIS with AF is consistent with existing evidence on stroke patients [37]. This finding indicates that stroke is not only a leading cause of death, but it is also a leading cause of disability including poor ambulatory outcome particularly in females in whom poor functional outcomes due to a stroke consistently exceeds male patients [38]. Our findings indicate that in AIS patients with AF, excess risk of stroke and poor prognosis may be caused by specific comorbidities and risk factors including migraines, higher HDL-cholesterol, and LDL-cholesterol in female AIS with AF. Migraineurs are known to present with increased levels of cholesterol, HDL-cholesterol, and LDL-cholesterol [39]. In addition, a large population-based cohort study found an increased odd of migraine among those with elevated total cholesterol. This effect was stronger among females that experience migraine than among males [40]. Moreover, females with higher total cholesterol present with an increased risk for AIS compared to females whose cholesterol was lower [31]. Increased risk of stroke is also associated with an elevated ratio of total cholesterol: HDL-cholesterol, or decreased HDL-cholesterol [41]. While previous studies have suggested an increased risk of AIS in males with a high total cholesterol: HDL ratio [42], the current study extends the possibility of HDL-cholesterol and LDL-cholesterol to female AIS patients with a baseline AF.

Therefore, our findings that in the AIS patients with AF, migraines, elevated HDL-cholesterol, elevated LDL-cholesterol, and poor ambulation were associated with females, while history of drug and alcohol abuse, sleep apnea, and increased serum creatinine level were associated with male AIS patients with AF supports our hypothesis that a gender based disparity in comorbidities and risk factors occurs in AIS population with baseline AF. While the frequencies of vascular disease vary between the males and females AIS patients, and the rates of hypertension (60% versus 56%) are higher in females than in males [33], we show that some risk factors including migraines, HDL-cholesterol, LDL-cholesterol maybe specific to female AIS patients with a baseline AF. Moreover, rates of drugs and alcohol abuse history, sleep apnea, and serum creatinine level were higher in male AIS with baseline AF compared to females.

The prevalence of stroke in the female gender is predicted to rise rapidly due to the continuous increase in the global elderly female population [38]. Our current findings support the possibility that specific baseline risk factors and or comorbidities may have significant effect in females than males, and this may contribute to the observed gender difference among stroke patients with baseline AF. Although our current data does not provide evidence for why a specific comorbidity and or risk factor may have a stronger effect on female AIS with AF than males, there are several possibilities. They include the possibility of under-treatment of females and physiological differences between the male and females [43, 44]. Stroke onset occurs later in age among females compared with males, and rates of AF(24% versus 22%) are higher in females than in males [45]. Atrial fibrillation is associated with a double the risk of stroke in females compared with the risk in males [43], and females with atrial fibrillation are known to present with more severe strokes than males [46]. Therefore, existing evidence together with our current findings support the possibility that specific comorbidity and or risk factors may have more effect in female than male stroke patients with baseline AF. In this context, the different burden of comorbidities and risk factors in both males and female AIS patients with AF needs to be thoroughly explored to personalize prevention and treatment.

There some limitations in the interpretation of the results of this study. This study was conducted at a single institution; therefore, the findings cannot be generalized to other institutions. Retrospective studies are known to have biases for selection because the data is not randomized. We predetermined all our subgroup analyses and repeated our analysis several times to eliminate the possibility of type 1 statistical errors. Our female subgroup analyses conducted reveal more significant variables associating more risk factors and comorbidities in females more than that of male AIS with AF. While this is a single study, the demonstration of consistent gender disparities in the AIS with and without AF increases the generalizability of our findings. Since mRS as a validated index of function for outcome was not included in the registry data based, therefore functional outcome could not be determined.

## Conclusion

Our findings reveal that migraines, elevated HDL, and elevated LDL were associated with female AIS patients with a baseline AF, while drugs and alcohol abuse history, sleep apnea, and higher serum creatinine level were associated with male AIS with baseline AF. Improved management strategies for baseline risk factors for AIS patients with a history of AF will be beneficial for both males and females AIS patients with AF.

## Data Availability

The retrospective datasets are available by request from the corresponding author of this manuscript respectively.

## Abbreviations

Adjusted OR: Adjusted odd ratio
Afib: Atrial fibrillation
HRT: Hormone Therapy
BMI: Body mass index
CHF: Congestive heart failure
CI: Confidence interval
IRB: Institutional Review Board
INR: International normalized ratio
LDL- C: Low-density lipoprotein cholesterol
HDL-C: High-density lipoprotein cholesterol
rtPA: Recombinant tissue plasminogen
TC: Total cholesterol
TG: Triglyceride
AIS: Acute ischemic stroke
NIHSS: National Institute of Health Stroke Scale
MRI: Magnetic Resonance Imaging
CT: Computer Tomography
MCA: Middle cerebral artery
CAD: Coronary artery disease
HRT: Hormone replacement therapy
TIA: Transient ischemic attack
PVD: Peripheral vascular disease
ROC: Receiver Operating Curve
INR: International Normalized Ratio
